# A linear programming approach for placement of applicants to academic programs

**DOI:** 10.1186/2193-1801-2-682

**Published:** 2013-12-19

**Authors:** Biniyam Asmare Kassa

**Affiliations:** College of Business & Economics, Bahir Dar University, Bahir Dar, Ethiopia

**Keywords:** Student placement, Linear programming, Management science, Educational planning

## Abstract

This paper reports a linear programming approach for placement of applicants to study programs developed and implemented at the college of Business & Economics, Bahir Dar University, Bahir Dar, Ethiopia. The approach is estimated to significantly streamline the placement decision process at the college by reducing required man hour as well as the time it takes to announce placement decisions. Compared to the previous manual system where only one or two placement criteria were considered, the new approach allows the college’s management to easily incorporate additional placement criteria, if needed. Comparison of our approach against manually constructed placement decisions based on actual data for the 2012/13 academic year suggested that about 93 percent of the placements from our model concur with the actual placement decisions. For the remaining 7 percent of placements, however, the actual placements made by the manual system display inconsistencies of decisions judged against the very criteria intended to guide placement decisions by the college’s program management office. Overall, the new approach proves to be a significant improvement over the manual system in terms of efficiency of the placement process and the quality of placement decisions.

## Introduction

The placement of applicants for undergraduate and graduate studies in various academic programs is one of the decision problems that administrators in universities regularly deal with. Given that applicants’ preferences for programs are not matched with capacities of available programs, administrators have used one or more criteria, along with applicants’ preferences, in an effort to best satisfy applicants’ interests while meeting other academic standards. In essence, the placement problem is a problem of allocation of limited intake capacities of academic programs or departments among applicants while trying to best satisfy the interest of applicants. When the placement decision involves hundreds of applicants using multiple criteria, it becomes a demanding problem to tackle manually.

This has been the experience at the College of Business and Economics of Bahir Dar University (CoBE hereafter) in recent years due to the increasing number of applicants. The current placement approach has become too laborious a placement approach, even when only one or two criteria are used to determine which applicants get placed in their more preferred programs of studies. The process is highly demanding in terms of staff time required to decide on placements. For instance, announcement of placement of more than 800 applicants to six undergraduate programs during the 2012/2013 academic year took about a week.

To rectify the situation, we developed a simple linear program based decision support system that can perform placement of applicants in a matter of a few seconds once the relevant data are entered. Though the model underlying our approach is related to models developed in related applications (Heckman and Taylor, 1969; Anwar and Bahaj, 2003; Pan et al., 2009), we deal with a much more simplified semi-assignment model (Pentico, 2007).

The new approach allows for consideration of a rich variety of placement criteria and improves consistency and clarity of the decision process, while boosting the efficiency of the placement decision process. The results of our model also provide useful insights such as about expansion of departmental intake capacities.

In this paper we present the placement model along with test results using actual placement data for the academic year 2012/13, and compare the results from our model with actual placement decisions. While our model makes identical placement decisions with actual placements for about 93 percent of applicants, the 7 percent differences of placements are shown to be the consequence of the inconsistency of the manually constructed placement decisions in a pattern that is easily visible. Specifically, the actual placements lack consistency in the case of applicants whose college entrance examination scores are in the bottom range and the marginal differences of the scores among applicants are relatively small.

The paper is organized as follows. In the next section a brief description of the problem is presented. In the penultimate section, we discuss the placement decision process at CoBE and present solution outputs based on actual data and compare the results from our model with actual placement decisions. Concluding remarks are provided in the last section.

## The placement model

### Notations

I: = set of applicants (i = 1,2,….,m)

J: = set of academic programs (j = 1,2,…,n)

K: = set of placement criteria (k = 1,2,…,r)

T: = set of cutoff points (t = 1,2,…,v)

G: = set of component objectives (g = 1,2,…,h)

Kt: = {k ϵ K: k = criterion for which cutoff point t ϵ T is applicable}

### Parameters

The parameters in our model are the following:

Uj: = maximum intake capacity of program j

Lj: = minimum number of applicants required to be placed in program j

sik: = applicant i’s score on criterion k (all criteria scores are converted to relative scores based on maximum raw criteria scores)

pij: = applicant i’s preference rating for program j (where the ratings in increasing preference order are p_ij_ = 1,2,…,n)

γjk: = minimum score on criterion k that is considered to be desirable for entry to program j

ωk: = weight assigned to criterion k in computing overall average score

qi: = overall average score of applicant 

dij: = a measure of the desirablity of assigning applicant i to program j = p_ij_ q_i_

βtk: = the value of criterion k ϵ K_t_ corresponding to cutoff point t ϵ T

θtk: = the preference value corresponding to cutoff point t ϵ T and criterion k ϵ K_t_

σg: = weight for component objective g

### Variables

xij: = equals 1 if applicant i is placed in program j and 0 otherwise.

### Constraints

There are two types of constraints: program capacity constraints and assignment requirements. Given the maximum intake capacity and minimum number of placements required per program we have the following constraints.1

Also, it’s required that an applicant gets placement in no more than one of the programs open for enrollment. This requirement is specified as:2

### Objective

Our objective function consists of four components each representing certain requirements specified by the program management office. The first component represents the desire to place applicants in programs of their higher preferences, with applicants’ overall average score determining priorities. This component is thus:3a

The second component of our objective function represents the desire to place applicants in their top rated programs. This component is formulated as:3b

In equation (3b), the variables are weighted by the applicants’ overall average score on all criteria. The aim is that assignment of applicants in their first rated programs be done with applicants with higher overall scores given higher priorities.

The third component represents the program placement office’s desire that applicants who have some specified scores on a certain criterion be placed in programs of their higher preference ratings (both the score and the preference rating cutoff points are decided by the office). Thus, for instance, assuming six open programs for enrollment, if it’s required that applicants who scored 350 and above marks on college entrance examination be placed in programs of their first (rating of 6) or second (rating of 5) preference, the criterion cutoff point is 350 and the preference cutoff point is 5.3c

The final and fourth component of our objective function represents the desire to minimize the cost of placements of applicants who fall short of the minimal requirements of entry specified in terms of minimum score on a criterion for a program. This is formulated as:3d

We combine these four components into a single objective function.3e

The model presented above is what Pentico (2007) refers to as a semi-assignment model, except for the multiple objective components. More generally, the model is a transportaiton model with applicants being sources and programs being recipients. Thus, our model can be solved as a linear program without explicitly imposing the binary restrictions on the decesion variables.

Our model is easily usable with many alternative placement criteria and weighting schema. Whatever the number of criteria and weighting schema used, however, college administrators should deliberate and agree on a set of objective and measurable criteria that will be used for placement of applicants in programs. The chosen criteria may depend on the objective of placement decisions.

Another contentious issue is the construction of the intake capacity parameters of the programs. Previously the capacity of departments was arbitrarily decided. This used to be the cause of conflicts among departments. Because students’ preference rankings tend to be skewed in favor of one or two programs, some political activity is involved to convince administrators to place applicants in programs even if that is against the interest of applicants. The real gap here is the absence of any standard that justifiably determines the number of students each program can enroll. Defining ex ante the criteria and formula for capacity determination will be of much help in this regard.

At CoBE, there are no formal mechanisms to determine the effective intake capacities of programs. In the case of placements for undergraduate programs reported below, the simple rule applied was that a minimum of a class size of applicants be placed in each of the six programs offered at CoBE. Satisfying this, any number of eligible applicants can be placed in a program. Though this is problematic in principle (for instance, suppose all applicants make one program their first choice), experience suggests this simple rule is usually adequate. Applicants’ preferences, though skewed towards a few programs, tend to be distributed across programs without causing too much imbalance between capacity and enrolment for any one program.

## Test results

In this section we present test results constructed with our model based on actual placement data for the 2012/13 academic year and compare the results with actual placement decisions. The placement decision support system we developed at CoBE is designed to support placement decisions both at undergraduate and graduate levels offered in any of the formats (regular, continuing, extension). However, the placement decision for the regular undergraduate programs has the largest problem size mainly due to the larger number of applicants in this format. For this reason, the system has found its greatest value in making placement decisions for the regular undergraduate programs. Currently, six undergraduate programs are offered in the regular program format. The experimental test results presented below concern placements made in these programs for the 2012/13 academic year at CoBE. The six undergraduate programs offered at CoBE are Accounting & Finance (ACT), Economics (ECO), Logistics & Supply Chain Management (LOG), Management (MGT), Marketing Management (MKT) and Tourism and Hotel Management (TOR). The experimental results are based on a study conducted as part of the development of the placement decision support system at CoBE and is officially approved by the academic commission (AC) of the College of Business & Economics of Bahir Dar University.

### The placement decision process

The process of placement at CoBE is briefly as follows. Every New Academic Year (the middle of September), students who have fulfilled the minimum requirements for college entry are assigned to broad study fields (such as humanities, engineering, business & economics, etc.) along with the specific universities where they pursue their studies by a body designated for this task at the Ethiopian Ministry of Education. The appropriate academic units within the universities then use their own criteria to place the students assigned to them in the various undergraduate study programs they offer. At CoBE only one criterion (aside from preferences of students) has been considered in placement of applicants in regular undergraduate programs: marks earned on college entrance examination by applicants. We are not suggesting here that applicants’ preference ratings for programs are not considered. Rather, applicants’ college entrance examination marks make the difference as to who gets placed in his or her more preferred program of study.

The placement decision process at CoBE is significantly an iterative process in which the value of even some of the attributes (such as capacity of programs) that would have been fixed a priori in our optimization model are also decision variables. For this reason distilling consistent principles underlying the process was difficult. Through discussions with the program manager of the college we were able to identify crude general guidelines underlying the actual placement decision process, as summarized below.

- The actual placement decisions were made on the basis of applicants’ marks which determine which students get placed in their more preferred programs.
- Getting applicants placed in the programs of their higher preference ratings, as far as possible, was considered the main objective.
- Applicants who scored 325 and above marks on the national college entrance examination must be placed in their most preferred programs, in spite of capacity and other considerations.
- For two applicants with the same preference rating for a given program, if one of them has a higher mark than the second, the applicant with higher marks should preempt the applicant with lower marks.
- For two applicants with different marks if the preference ratings of the applicants for a given program are also different, for the program under consideration the applicant with lower marks may preempt the applicant with higher marks only if the applicant with lower marks has given a higher preference rating for the program than did the applicant with higher marks. Thus, in this case there is substitution between marks and preference.
- A minimum of a class size of applicants must be placed in each of the six programs (where a class size roughly equals 50 students)

As will be shown below, the actual placement decisions were largely consistent with regard to these guidelines. Nonetheless, about 7 percent of actual placements showed inconsistencies.

### Data

#### Applicants’ program preferences

Conversion of preference rankings to preference points was necessary to make the preference rankings of applicants for the six programs suitable to our analysis. Applicants have filled out a form indicating their choices of programs by simply writing their preference ranks for their top three preferred programs. We converted these into numerical values. Our preference rating schema assigns rating points of 6, 5 and 4 for the first, second, and third choices of programs, respectively. Since applicants were not required to rank all programs for their preferences, if a program is not in the top three preference list of an applicant, we have no data about the applicant’s preference ranking for this program. For this reason we assumed equal rankings (with a preference rating value of 3) for the remaining three programs that are not included in an applicant’s top three preference list. The distribution of applicants’ preference rankings for the programs is summarized in Table 1.

**Table 1:** **Distribution of applicants by their program preferences**

Program	No. of applicants who rate a program as their				
	First choice	Second choice	Third choice	Total	Percent
ACT	330	234	105	669	82%
ECO	177	173	198	548	67%
LOG	12	33	55	100	12%
MGT	209	240	247	696	86%
MKT	51	92	131	274	34%
TOR	33	39	75	147	18%
Grand Total	812	811*	811*		

*One applicant indicated only his first preferred program (ACT) without rating the other programs for his preference. For this student, we assumed equal ratings of 3 for all the remaining programs.

The preferences are concentrated in favor of three programs - ACT, ECO and MGT. A better way to understand the preferences of applicants for the six programs is based on our preference point assignments. Note that if a program is the first choice of all 812 applicants, the program’s aggregate preference points will be 4872. Thus, we can compare preferences of applicants for programs relative to this maximum possible aggregate preference value (Table 2).

**Table 2:** **Aggregate preference points for programs**

	ACT	ECO	LOG	MGT	MKT	TOR
Actual aggregate preferences of all applicants	3999.00	3511.00	2593.00	3790.00	2904.00	2688.00
Hypothetical Aggregate preference value of program	4872.00	4872.00	4872.00	4872.00	4872.00	4872.00
Actual as percentage of hypothetical (%)	82	72	53	78	60	55
Average preference level for the program	4.92	4.32	3.19	4.67	3.58	3.31

The figures in Table 2 again confirm that ACT, ECO and MGT are the top three preferred programs by applicants. It is such unbalanced distribution of applicants’ preferences, when considered against limited intake capacities of programs, that gives meaning to the scarce resource allocation view of placement decisions in universities. To the extent that the capacities of the programs that are highly rated by many applicants are limited and can’t accommodate all applicants, it’s necessary that placement decisions be made to best ‘utilize’ the limited intake capacities of the programs.

#### College entrance examination scores of applicants

Marks earned by applicants in the national college entrance examination are the main criteria used in the actual placement decision. Table 3 summarizes the distribution of the marks earned by applicants.

**Table 3:** **Distribution of scores on college entrance examination by program of actual placement**

Program of actual placement	Average	Maximum	Minimum	SD
ACT	366.63	475.00	314.00	27.61
ECO	376.11	498.00	328.00	36.47
LOG	321.33	356.00	312.00	10.06
MGT	353.41	469.00	317.00	24.77
MKT	334.65	420.00	312.00	23.53
TOR	330.67	400.00	312.00	19.24
Overall	357.53	498.00	312.00	32.14

#### Capacity of programs

For the sake of comparability, for the results reported here we take the actually assigned number of applicants in each program as that program’s intake capacity. Hence, the capacities of the six programs under consideration are the number of students actually placed in them as shown in Table 4.

**Table 4:** **Capacity of programs/actually placed number of students**

Program	Capacity/Actually placed no of students
ACT	284
ECO	165
LOG	54
MGT	190
MKT	71
TOR	48
Grand Total	812

### Results and discussion

The linear programming placement model was written into a text file. The model and command codes as well as the data are read by AMPL which also provides pre-solve routines which process the model before sending to the optimizer (Fourer et al., 2003). When the pre-solve stage is completed, the Gurobi.5.5.0 linear optimizer implements its algorithm to find an optimal solution to the model. It took no more than 5 seconds to obtain optimal solutions for the test problem presented here.

Table 5 displays a comparison of actual placements and placements based on the solutions for our model.

**Table 5:** **Placements and applicants’ preferences, actual and model results**

Applicants’ preference ranking	Number placed		% Placed		Cumulative %	
	Model	Actual	Model	Actual	Model	Actual
1st	735	720	90.52	88.67	90.52	88.67
2nd	26	34	3.20	4.19	93.72	92.86
3rd	14	22	1.72	2.71	95.44	95.57
Last	37	36	4.56	4.43	100	100
Total	812	812	100	100		

About 89 percent of applicants were actually placed in a program of their first preference and 4.2 percent of applicants in a program of their second preference. Equivalently, our model has placed 90.5 percent of applicants in a program of their first preference and 3.2 percent of applicants in a program of their second preference. Thus, both systems have placed about 93 percent of applicants in a program of their first or second preference. This suggests that with respect to applicants’ preferences our model generates at least as much quality output as the manual system (Table 6).

**Table 6:** **Summary of actual placements by similarity with model placements**

Program of actual placement	Actual and model placements identical			Actual and model placements different			Overall summary		
	Average (Marks)	SD (Marks)	No. placed	Average (Marks)	SD (Marks)	No. placed	Average (Marks)	SD (Marks)	No. placed
ACT	367.4	27.3	278	329.8	14.5	6	366.6	27.6	284.0
ECO	376.4	36.4	164	328.0	na	1	376.1	36.5	165.0
LOG	322.5	11.1	42	317.3	2.7	12	321.3	10.1	54.0
MGT	355.4	24.6	174	331.4	14.1	16	353.4	24.8	190.0
MKT	338.4	24.8	57	319.4	3.6	14	334.6	23.5	71.0
TOR	331.8	19.7	44	318.0	3.6	4	330.7	19.2	48.0
Grand Total	359.9	31.8	759	323.8	11.1	53	357.5	32.1	812.0

But, a deeper investigation allows us to learn that the placements from our model have in some sense an even better quality than the actual placements. To see this, first consider the differences and similarities of the placements according to the two approaches. As shown in Table 6, the two approaches make 759 identical placements which is equivalent to 93 percent of the placements. Only in the remaining 7 percent of placements (53 placements) do the two differ.

Now setting aside the identical placements, we consider those for which the two systems differ in their placement decisions. One should first note, as discussed, that the intended guiding principle of the actual placement decision process at CoBE was to maximize the number of applicants placed in their more preferred programs while applicants’ marks serve to prioritize identical preferences of two or more applicants competing for the same program. Also, the actual decision process was meant to ensure appropriate tradeoffs between preferences and marks. According to the program management office, the belief was that if an applicant is outcompeted for a program of his or her first preference, the applicant will be considered for placement in a program of his or her second preference. If there is still competition, tradeoffs between marks and preferences will be considered: specifically, if, for this student’s program of second preference, applicants with lower marks are also competing, this applicant may not be placed in the program of his or her second preference if the applicants’ with lower marks than this student’s have a higher preference rating for the same program. This is the tradeoff principle we mentioned above. Finally, there should not be any obvious misplacement. That is, the placement of applicants based on applicants’ preferences and marks should do one thing: whenever two applicants have the same preferences but one applicant’s marks are greater than the other applicant, the applicant with higher marks should be placed in that program. We will discuss the extent to which these principles are adhered to. Table 7 provides the necessary information for this.

**Table 7:** **Placements, marks and preferences based on actual placements that differ from model placements**

	Preference of applicants not placed = Preference of applicants placed		Preference of applicants not placed > Preference of applicants placed	
Marks of applicants placed = marks of applicants not placed	Actual = 3	II	Actual = 6	I
	Model = 3		Model = 0	
Marks of applicants placed < marks of applicants not placed	Actual = 39	III	Actual = 5	IV
	Model = 0		Model = 0	

Quadrant II represents cases for which any placement is as good as another because both preferences and criteria scores are equal for the applicants under consideration (three of the differences between the actual and model placements belong to this category). Quadrant I is the number of placements such that applicants placed and applicants not placed in a given program have the same marks but those not placed ranked that program higher than those placed. Indicated in quadrant III and quadrant IV are number of applicants for which placements in a program seem arbitrary in that when two applicants have different marks and both choose a program equally (quadrant III) or the applicant with higher marks also has stronger preference for the program (quadrant IV), the actual placement gives priority to the applicant with lower marks, which is against the very guiding principles discussed above.

Looking back again to Table 6 we may be able to explain why the manual placement system compares poorly to our model in this case. The first thing to note from the table is that the average mark of students whose placements are the same in both approaches is significantly (in the statistical sense of the term) different from the average mark of applicants whose placements are different using the two systems. Thus, it is highly likely that the placement decision makers took their time to ensure that the top scoring students got placed in their place. After some cutoff point of marks, they then took it that for applicants scoring marks below this point which program they got assigned to is immaterial. Or, these students are likely to be content with whichever program they got assigned to. In a sense, the placement personnel may find it unnecessarily wasteful of their time to dwell on careful placement of these students.

An even more interesting insight as to what might have gone into the actual placement process is suggested when we consider the standard deviations of the marks for the two groups of placements. The standard deviation of marks is consistently smaller, department by department, for those placements on which the actual and our model’s placements differ. The same holds true when we also consider the overall standard deviation for the two groups. The placements on which our model and the manual system don’t agree have a significantly lower standard deviation. But this may indicate that for applicants with lower marks, the decision makers were supposed to concern themselves not only with lower marks but also marginally smaller differences of marks among these students. This makes the task even more difficult because smaller margins are much more difficult to assess. Thus, this adds to the likelihood that decisions of placements for applicants with lower marks are apt to be inaccurate.

In contrast, the coefficients in our model’s objective function easily handle such requirements. This makes our model even more compelling (Table 8).

**Table 8:** **Comparison of actual and model placements according to aggregate preference ratings**

Comparison criterion	Model	Actual
Applicants’ total preference values for programs of their placement	4743	4686
Hypothetical aggregate preferences of applicants (if all applicants were placed in their top preferred programs)	4872	4872
Percent relative to hypothetical aggregate preference value (%)	97	96

Finally, looking at Table 8 one can learn that the aggregate preferences of applicants to programs of their actual placements is slightly less than the aggregate preferences achievable using our model. Note here that had all applicants been placed in their first preferred programs, the aggregate preferences would have been 4872 points. Comparisons can be made relative to this maximum possible value. Thus, our placement model achieves an aggregate preference total of 4743 which is about 97 percent of the maximum possible value. By comparison, only 4686 points, which is about 96 percent of the maximum possible points, were achieved in the actual placement decisions. Thus our model consistently prioritizes applicants by their marks while achieving slightly higher level of aggregate preference points - which means better level of aggregate satisfaction of applicants relative to their actual placements.

## Conclusion

In this paper we presented a linear programming placement model that is already approved and to be applied for actual placement decisions starting with this academic year at the college of business and economics, Bahir Dar University. The test results based on actual placement data indicated that our model proves to produce at least as much quality of output as the manual system. Nonetheless, in a deeper sense, our model is even superior as it can be easily adjusted to strike a desirable tradeoff between the various criteria used to decide on placements. All these gains, when considered along with the significant efficiency gains that our placement model entails, suggest the potential value of management science in improving decision making in Ethiopian higher learning institutions. We hesitate little to encourage adoption of our model in other similar institutions in the country.

## Consent

Written informed consent was obtained from the program manager of the College of Business & Economics for the publication of this report and any accompanying information.
